# Bexarotene Reduces Blood-Brain Barrier Permeability in Cerebral Ischemia-Reperfusion Injured Rats

**DOI:** 10.1371/journal.pone.0122744

**Published:** 2015-04-06

**Authors:** Lu Xu, Fang Cao, Feng Xu, Baicheng He, Zhi Dong

**Affiliations:** Chongqing Key Laboratory of Biochemistry and Molecular Pharmacology, College of Pharmacy, Chongqing Medical University, Chongqing 400016, China; Emory University School of Medicine, UNITED STATES

## Abstract

**Background:**

Matrix metalloproteinase-9 (MMP-9) over-expression disrupts the blood-brain barrier (BBB) in the ischemic brain. The retinoid X receptor agonist bexarotene suppresses MMP-9 expression in endothelial cells and displays neuroprotective effects. Therefore, we hypothesized that bexarotene may have a beneficial effect on I/R-induced BBB dysfunction.

**Methods:**

A total of 180 rats were randomized into three groups (n = 60 each): (i) a sham-operation group, (ii) a cerebral ischemia-reperfusion (I/R) group, and (iii) an I/R+bexarotene group. Brain water content was measured by the dry wet weight method. BBB permeability was analyzed by Evans Blue staining and the magnetic resonance imaging contrast agent Omniscan. MMP-9 mRNA expression, protein expression, and activity were assessed by reverse transcription polymerase chain reaction, Western blotting, and gelatin zymography, respectively. Apolipoprotein E (apoE), claudin-5, and occludin expression were analyzed by Western blotting.

**Results:**

After 24 h, 48 h, and 72 h post-I/R, several effects were observed with bexarotene administration: (i) brain water content and BBB permeability were significantly reduced; (ii) MMP-9 mRNA and protein expression as well as activity were significantly decreased; (iii) claudin-5 and occludin expression were significantly increased; and (iv) apoE expression was significantly increased.

**Conclusions:**

Bexarotene decreases BBB permeability in rats with cerebral I/R injury. This effect may be due in part to bexarotene’s upregulation of apoE expression, which has been previously shown to reduce BBB permeability through suppressing MMP-9-mediated degradation of the tight junction proteins claudin-5 and occludin. This work offers insight to aid future development of therapeutic agents for cerebral I/R injury in human patients.

## INTRODUCTION

The activation and up-regulation of matrix metalloproteinase-9 (MMP-9) in the ischemic brain can lead to brain edema and hemorrhagic transformation through disrupting the blood-brain barrier (BBB) [1]. Furthermore, reperfusion with recombinant tissue plasminogen activator (tPA) can sometimes produce catastrophic hemorrhagic transformation in the ischemic brain by triggering MMP-9 activation [2]. On this basis, therapeutic targeting that inhibits MMP-9 activity may be a promising approach to minimizing secondary brain injury in acute stroke patients.

To this end, bexarotene (LGD1069) is a selective retinoid X receptor (RXR) agonist currently used in treating cutaneous T-cell lymphoma that has been shown to suppress MMP-9 expression in endothelial cells [3,4]. Notably, bexarotene is a fat-soluble small molecular weight (348.48 Da) agent that readily penetrates the BBB and displays neuroprotective effects. In particular, oral administration of bexarotene in a murine model of Alzheimer’s disease has been shown to enhance clearance of soluble amyloid β (Aβ) peptide in an apolipoprotein E (apoE)-dependent manner while improving cognitive, social, and olfactory deficits [5].

Despite these promising findings, the effects of bexarotene upon acute brain injury have not been thoroughly investigated. In order to explore the neuroprotective effects of bexarotene under cerebral ischemic-hypoxic conditions, here we constructed a cerebral I/R rodent model and assessed the effects of bexarotene upon brain water content, BBB permeability, MMP-9 expression and activity, tight junction integrity via claudin-5 and occludin expression, and apoE expression.

## MATERIALS AND METHODS

### Materials

A total of 180 male SD rats weighing 250±20 g in a SPF grade were obtained from the Experimental Animal Center of Chongqing Medical University (Chongqing, China). Bexarotene and EB stain were purchased from TakaRa Co. (USA). The MRI contrast agent Omniscan was purchased from GE Healthcare (China). Trizol, MMLV-RT, and the PCR kit were purchased from TakaRa Co. (Japan). Mouse anti-actin, rat anti-apoE, rabbit anti-MMP-9, rabbit anti-claudin-5, and rabbit anti-occludin antibodies were purchased from Cell Signaling Technology Co. (USA). All treatments were performed under sodium pentobarbital anesthesia, which was chosen for its safety, short onset time, long duration of effect, rapid recovery time, and low-cost. All efforts were made to minimize animal suffering. The Committee on Ethics of Animal Experimentation at Chongqing Medical University (Chongqing, China) approved the protocols of this study prior to its implementation (approval no.: 2013026).

### Construction of Experimental Groups and Cerebral Ischemia-Reperfusion Model

A total of 180 rats were randomly separated into three groups of 60 rats each: a sham-operation group, a cerebral I/R group, and an I/R+bexarotene group. Referring to Longa’s method [6], the cerebral I/R rodent model was constructed using the suture method. The left common carotid artery (CCA), the external carotid artery (ECA), and the internal carotid artery (ICA) were separated, and the ECA was ligated. Near the bifurcation of the CCA, a small “V” incision was performed. Approximately 1.8±0.2 cm nylon wire was inserted through the incision into the ICA. In the sham-operation subjects, nothing was inserted. At 2 h post-cerebral ischemia, the nylon wire was taken out to allow reperfusion. After reviving the rats, neurological function deficits were scored, and the experimental animals scoring from 1 to 3 were selected.

**Fig 1:**
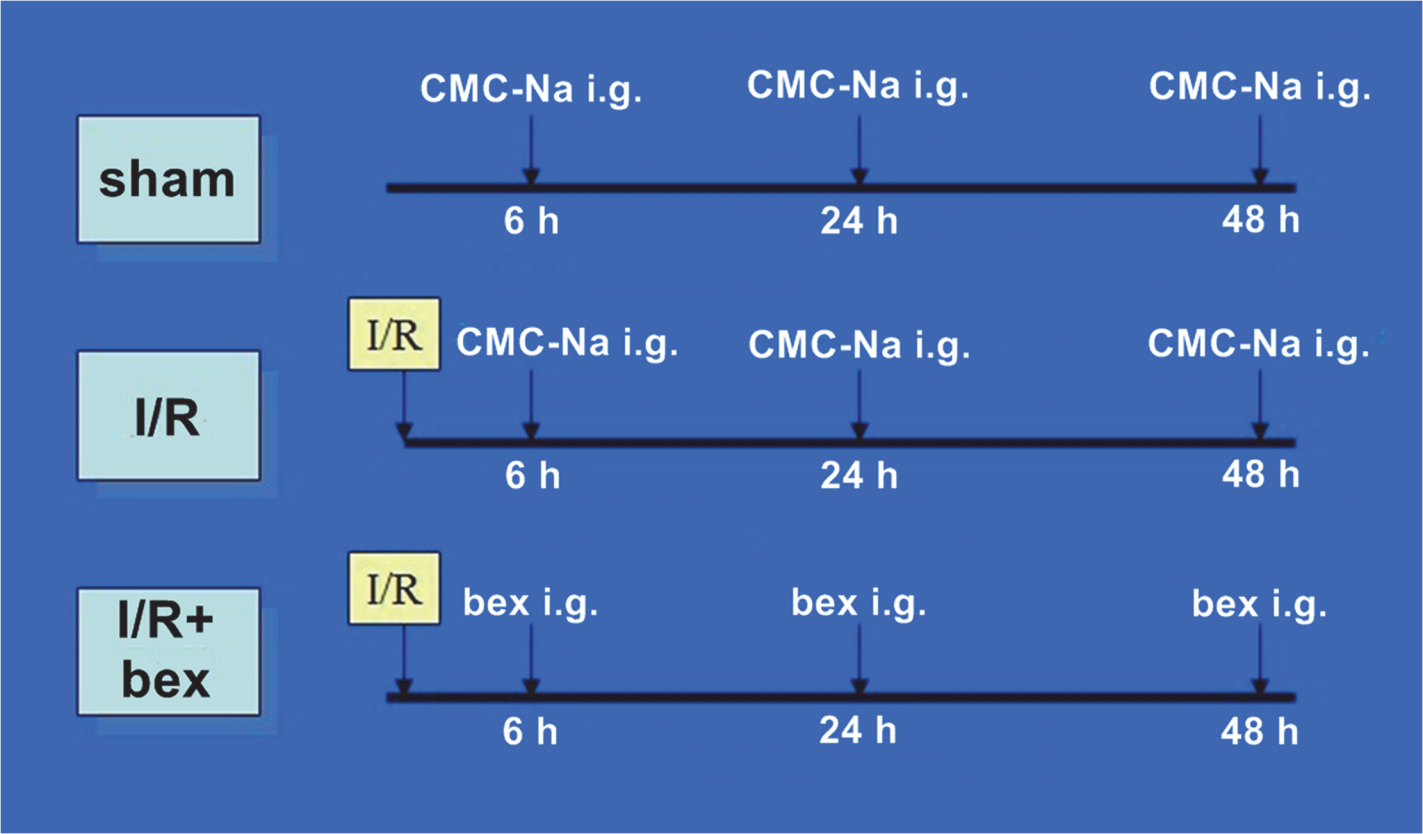
Schematic of Experimental Protocol.

A bexarotene suspension was prepared by dissolving bexarotene in 0.5% sodium carboxyl methyl cellulose (CMC-Na) at a concentration of 10 mg/ml. In 6 h, 24 h, and 48 h post-reperfusion, bexarotene (50 mg/kg·d) was given by intragastric gavage (i.g.); the sham-operation and I/R groups were given a corresponding volume of 0.5% CMC-Na (Fig 1). Ten rats in each group were sacrificed at 12 h, 24 h, 48 h, and 72 h post-reperfusion, respectively, five of which were used to measure BBB permeability and five of which were used for PCR, Western blotting, and gelatin zymography.

### Measurement of the Brain Water Content

At 12 h, 24 h, 48 h, and 72 h after I/R, five rats in each group were sacrificed under 3% sodium pentobarbital by intraperitoneal injection. To prevent evaporation, the brain was removed and immediately placed on saline-soaked filter paper. Blood and pia mater were carefully removed. Tissue wet weight was weighed (accurate to 0.1 mg), and then the brain tissue was placed in a drying oven (100°C±2°C). After 48 h, the dry weight was weighed (accurate to 0.1 mg). Brain water content was calculated according to the Blliot formula: BWC = (wet weight—dry weight)/wet weight×100%.

### Magnetic Resonance Imaging (MRI)

At 12 h, 24 h, 48 h, and 72 h after I/R, the MRI contrast agent Omniscan (1.5 ml/kg) was injected via caudal vein by a 1-ml syringe, and an advanced 1.5T nuclear magnetic resonance system (Siemens) was used to detect and evaluate BBB disruption. The head of the rat was caught by a special MRI signal enhanced coil with a 2-cm diameter. Precise MRI information was obtained from T1-weighted intensifier scanning. MRI signal data were collected by the MRI system software.

### Evans Blue (EB) Method

One hour prior to sacrifice, five rats in each group were injected with 2% EB (3 ml/kg or 60 μg/g) via the caudal vein. At 12 h, 24 h, 48 h, and 72 h after I/R, infusion with heparinized saline through the left ventricle was performed until colorless infusion fluid was obtained from the right atrium; then, the rats were decapitated. The right hemisphere was weighed, placed in a tube with 3 ml dimethylformamide (DMF), and then thawed in water at 60°C in a dark room for 24 h. The optical density (OD) value of EB was measured by a RF-540 fluorescence spectrophotometer (λ = 632 nm). The EB content was calculated from a standard EB curve to measure the change in BBB permeability.

### RT-PCR Detection of MMP-9 mRNA Expression

After MRI detection, five rats in each group were decapitated. The ischemic hemispheres were randomized into two parts: one for extracting total RNA according to the Trizol kit instructions and another for Western blot and gelatin zymography. The MMLV-RT kit was used to reverse transcribe total RNA into cDNA. The MMP-9 primer (5-primer 5’-AAA TGT GGG TGT ACA CAG GC-3’, 3-primer 5’-TTC ACC CGG TTG TGG AAA CT-3’, the amplified fragment was 310 bp in length) and the internal reference β-actin primer (5-primer 5’-ATC CTG CGT CTG GAC CTGG-3’, 3-primer 5’-TTG GCA TAG AGG TCT TTA CGG AT-3’, the amplified fragment was 365 bp in length) were amplified by PCR. In a 25 μl reaction volume, PCR was performed as follows: initial denaturation for 4 min at 94°C, followed by denaturation for 30 s at 94°C, annealing for 30 s at 62°C, and extension for 2 min at 72°C for 35 cycles, and finally extension for 10 min at 72°C. Electrophoresis was used to separate the PCR-amplified products, and the results were imaged and analyzed by a gel image analysis system. The quantitative index of MMP-9 mRNA expression was the ratio of the absorbance values of the PCR-amplified products to those of β-actin products.

### Gelatin Zymography Assay for MMP-9 Activity

MMP-9 activity was assessed by gelatin zymography assay. Tissue from the frontal and parietal lobes in the cerebral ischemic region was applied with cell lysate to extract the total protein (i.e., the ABC protein concentration method). Then, a 30-μg protein sample was separated by 12% sodium dodecyl sulfate polyacrylamide gel electrophoresis (SDS-PAGE) with 1% gelatin. After electrophoresis, SDS was eluted by incubating the gel in eluate containing 2.5% Triton X-100 for 20 min thrice, and Triton X-100 was then washed out by motion. The gel was incubated in substrate buffer for 48 hours at 37°C. After incubation, the gel was stained with 0.5% Coomassie Brilliant Blue solution for 4 hours. The gel was placed into a bleaching gel solution to destain until the digested band appeared, and then the gel was photographed for analysis.

### Western Blotting of ApoE, MMP-9, Claudin-5, and Occludin Protein Expression

Tissue from the frontal and parietal lobes in the cerebral ischemic region was applied with cell lysate to extract the total protein (i.e., the ABC protein concentration method). Then, a 30-μg protein sample was separated by 12% SDS-PAGE then transferred to a PVDF membrane. The PVDF membrane was blocked in 5% skim milk for 2 h and probed with the relevant primary antibody (1:200) for 1 h, cultured in 5% CO_2_ incubator at 4°C overnight, then incubated with a secondary antibody for 2 h. The Western blot fluorescence detection kit revealed a brownish red stripe for the protein of interest, and the gel image analysis system showed the density by photograph and scan. The internal reference was β-actin.

### Statistical Analysis

Measurement data were expressed as means±SDs and were processed by SPSS v20. Data from the same time points between different groups were compared by *t*-test, while data across different time points within the same group were compared with two-way ANOVA. *P*<0.05 was deemed statistically significant.

## RESULTS

### Brain Water Content Findings

**Fig 2:**
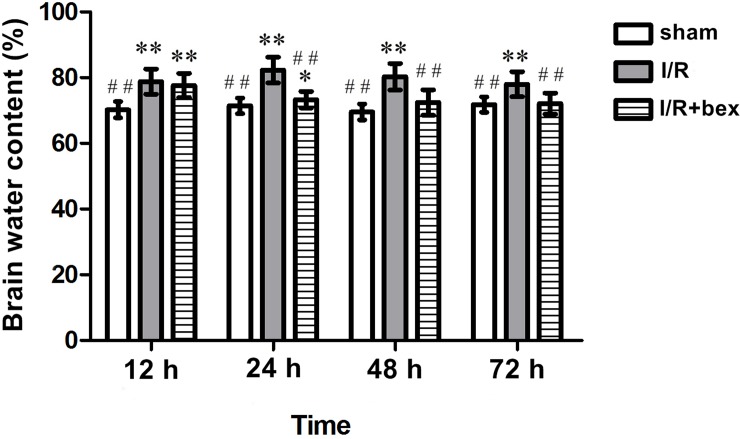
Brain Water Content. Brain water content was measured by the dry wet weight method. Values are expressed in % as means±SDs (n = 5 per group; *P<0.05 versus the sham group, **P<0.01 versus the sham group; #*P*<0.05 versus the I/R group, and ##*P*<0.01 versus the I/R group).

At 12 h, 24 h, 48 h, and 72 h post-I/R, brain water content was significantly increased in the I/R group as compared with the sham-operation group (*P*<0.01; Fig 2). At 12 h and 24 h post-I/R, brain water content was significantly increased in the I/R+bexarotene group as compared with the sham-operation group (12 h: *P*<0.01; 24 h: *P*<0.05, Fig 2). At 24 h, 48 h, and 72 h post-I/R, brain water content was significantly decreased in the I/R+bexarotene group as compared with the I/R group (*P*<0.01), while there was no significant difference at 12 h (*P*>0.05; Fig 2).

### MRI Findings

**Fig 3:**
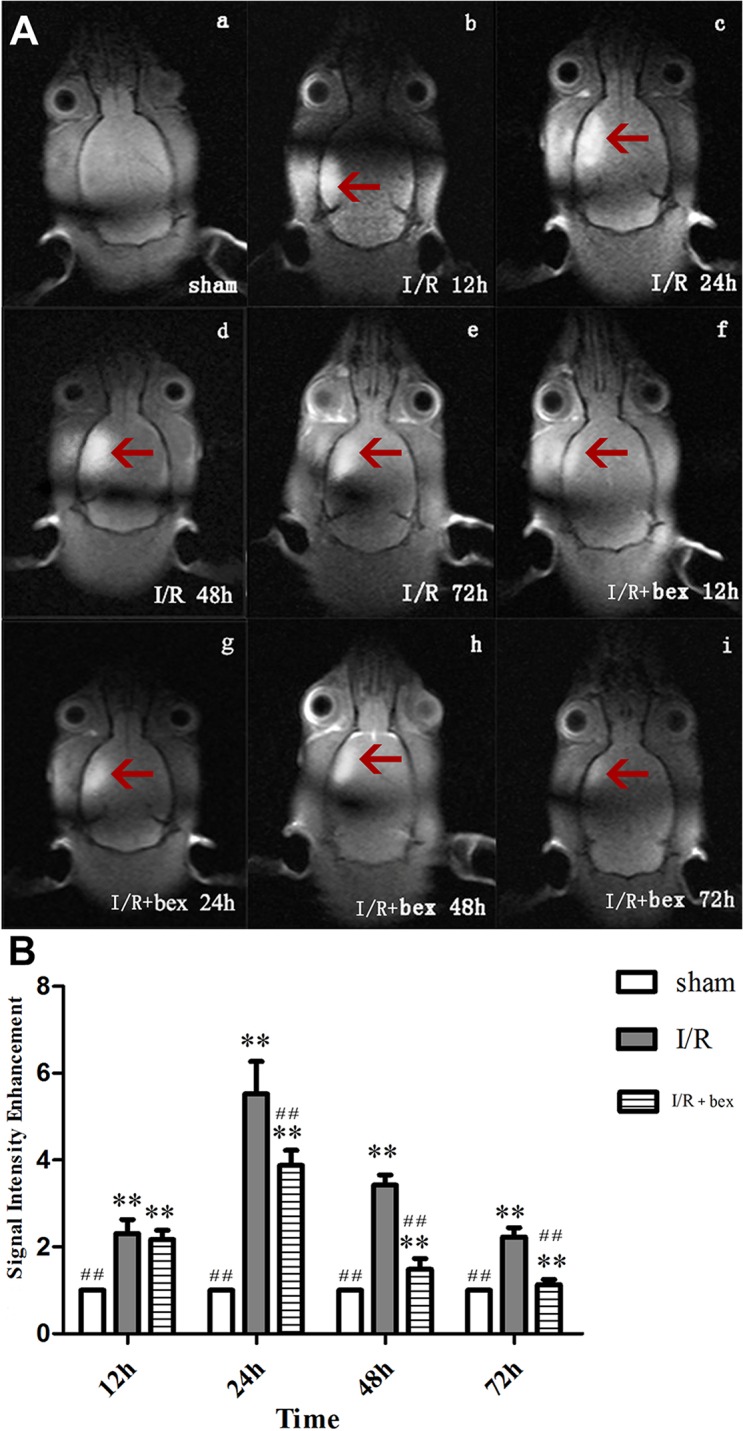
T1-Weighted Magnetic Resonance Imaging (MRI). Representative MRI images from the (a) sham group, (b) I/R group (12 h after I/R), (c) I/R group (24 h after I/R), (d) I/R group (48 h after I/R), (e) I/R group (72 h after I/R), (f) I/R+bexarotene group (12 h after I/R), (g) I/R+bexarotene group (24 h after I/R), (h) I/R+bexarotene group (48 h after I/R), and (i) I/R+bexarotene group (72 h after I/R). The red arrows point to the signal intensity-enhanced regions with larger regions and more pronounced signal intensities indicating more serious brain damage. (B) MRI signal values at each time point are expressed as means±SDs (n = 5 per group; *P<0.05 versus the sham group, **P<0.01 versus the sham group; #*P*<0.05 versus the I/R group, and ##*P*<0.01 versus the I/R group).

At 12 h, 24 h, 48 h, and 72 h post-I/R, significantly enhanced regions were found in both the I/R and I/R+bexarotene groups relative to the sham-operation group (*P*<0.01; Fig 3). At 24 h, 48 h, and 72 h post-I/R, the signal value was significantly decreased in the I/R+bexarotene group as compared with the I/R group (*P*<0.01), while there was no significant difference at 12 h (*P*>0.05; Fig 3).

### Evans Blue (EB) Findings

**Fig 4:**
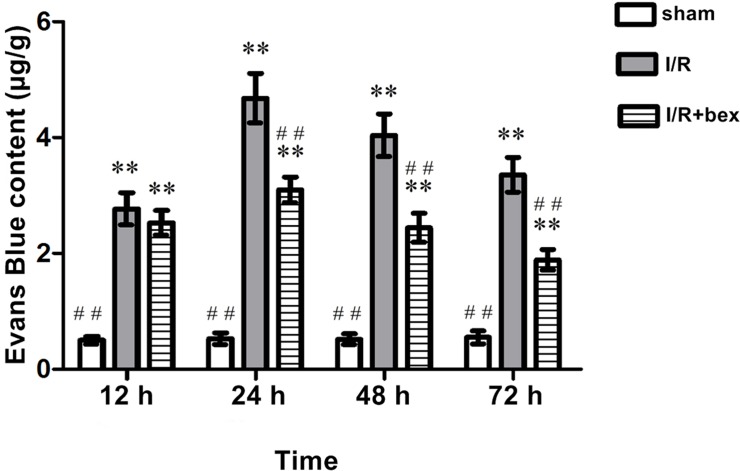
Evans Blue (EB) Method. Increased EB content in brain tissue confirms increased BBB permeability post-I/R. Values are expressed in μg/g as means±SDs (n = 5 per group; *P<0.05 versus the sham group, **P<0.01 versus the sham group; #*P*<0.05 versus the I/R group, and ##*P*<0.01 versus the I/R group).

At 12 h, 24 h, 48 h, and 72 h post-I/R, EB content was significantly increased in the I/R and I/R+bexarotene groups as compared with the sham-operation group (*P*<0.01; Fig 4). At 24 h, 48 h, and 72 h after reperfusion, EB content was significantly decreased in the I/R+bexarotene group as compared with the I/R group (*P*<0.01), while there was no significant difference at 12 h (*P*>0.05; Fig 4).

### MMP-9 mRNA Expression

**Fig 5:**
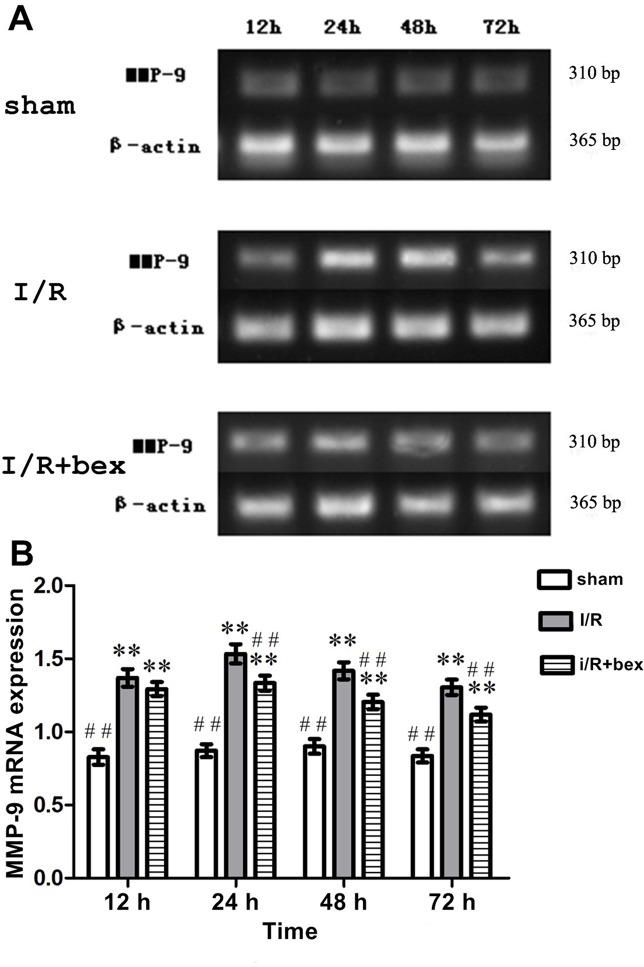
MMP-9 mRNA Expression. MMP-9 mRNA expression level in the ischemic zone of the rat brain was determined by RT-PCR with β-actin used as an internal control. (B) Densitometric analysis. Values are expressed as means±SDs (n = 5 per group; *P<0.05 versus the sham group, **P<0.01 versus the sham group; #*P*<0.05 versus the I/R group, and ##*P*<0.01 versus the I/R group).

At 12 h, 24 h, 48 h, and 72 h post-I/R, MMP-9 mRNA expression was increased in both the I/R and I/R+bexarotene groups as compared with the sham-operation group (*P*<0.01) with the highest expression achieved at 24 h (Fig 5). At 24 h, 48 h, and 72 h post-I/R, MMR-9 mRNA expression in the I/R+bexarotene group was lower than that of the I/R group (*P*<0.01), while there was no significant difference at 12 h (*P*>0.05; Fig 5).

### MMP-9 Activity

**Fig 6:**
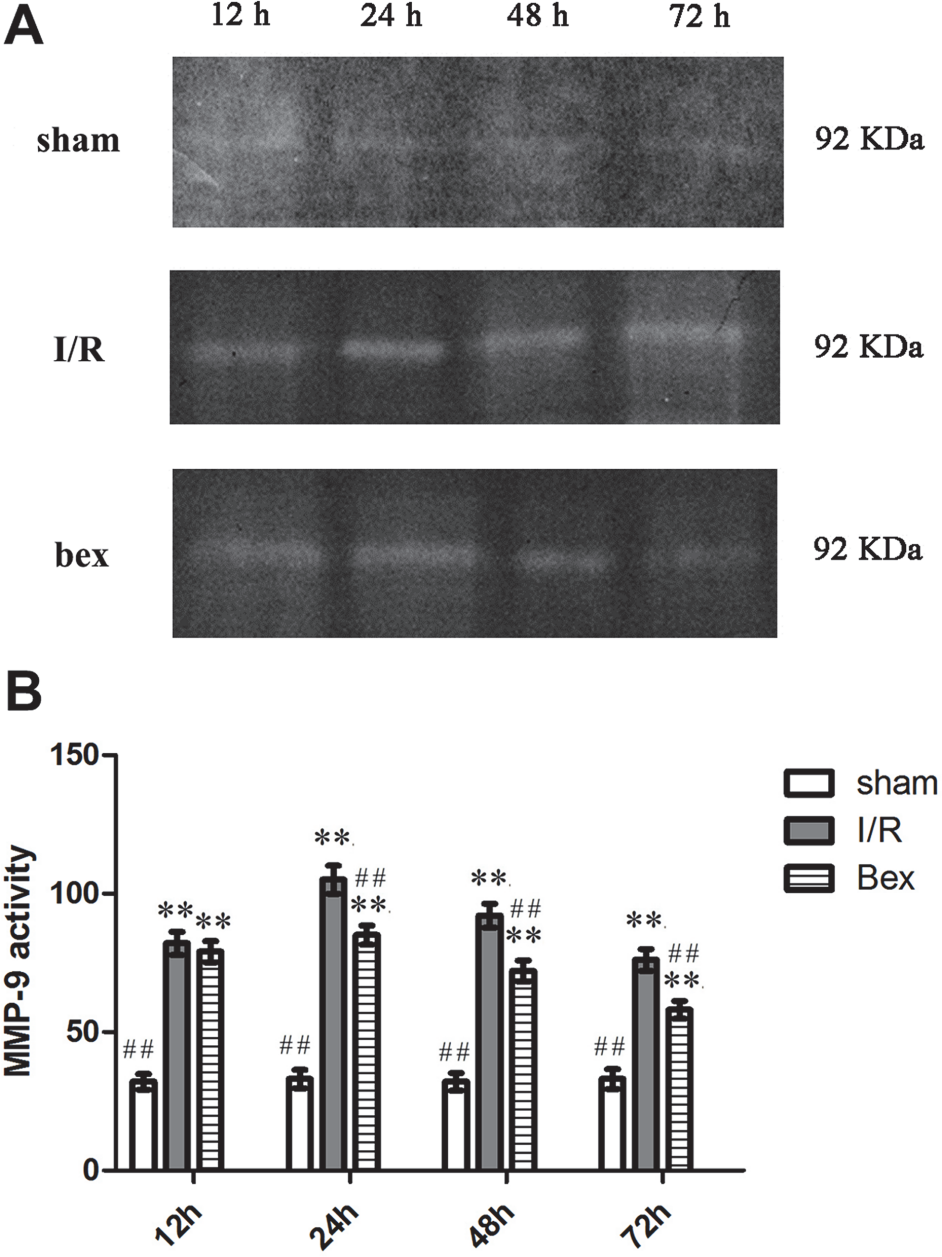
MMP-9 Activity. (A) MMP-9 activity in the ischemic zone of the rat brain was determined by gelatin zymography. (B) Densitometric analysis. Values are expressed as means±SDs (n = 5 per group; *P<0.05 versus the sham group, **P<0.01 versus the sham group; #*P*<0.05 versus the I/R group, and ##*P*<0.01 versus the I/R group).

Gelatin zymography analysis revealed a small amount of MMP-9 activity in the sham-operation group (Fig 6). In contrast, at 12 h, 24 h, 48 h, and 72 h post-I/R, MMP-9 enzyme activity was significantly increased in both the I/R and I/R+bexarotene groups as compared with the sham-operation group (*P*<0.01) as evidenced by the 92 KDa bands (Fig 6). At 24 h, 48 h, and 72 h post-I/R, MMP-9 activity was significantly lower in the I/R+bexarotene group as compared to the I/R group (*P*<0.01), while there was no significant difference at 12 h (*P*>0.05; Fig 6).

### ApoE, MMP-9, Claudin-5, and Occludin Protein Expression

**Fig 7:**
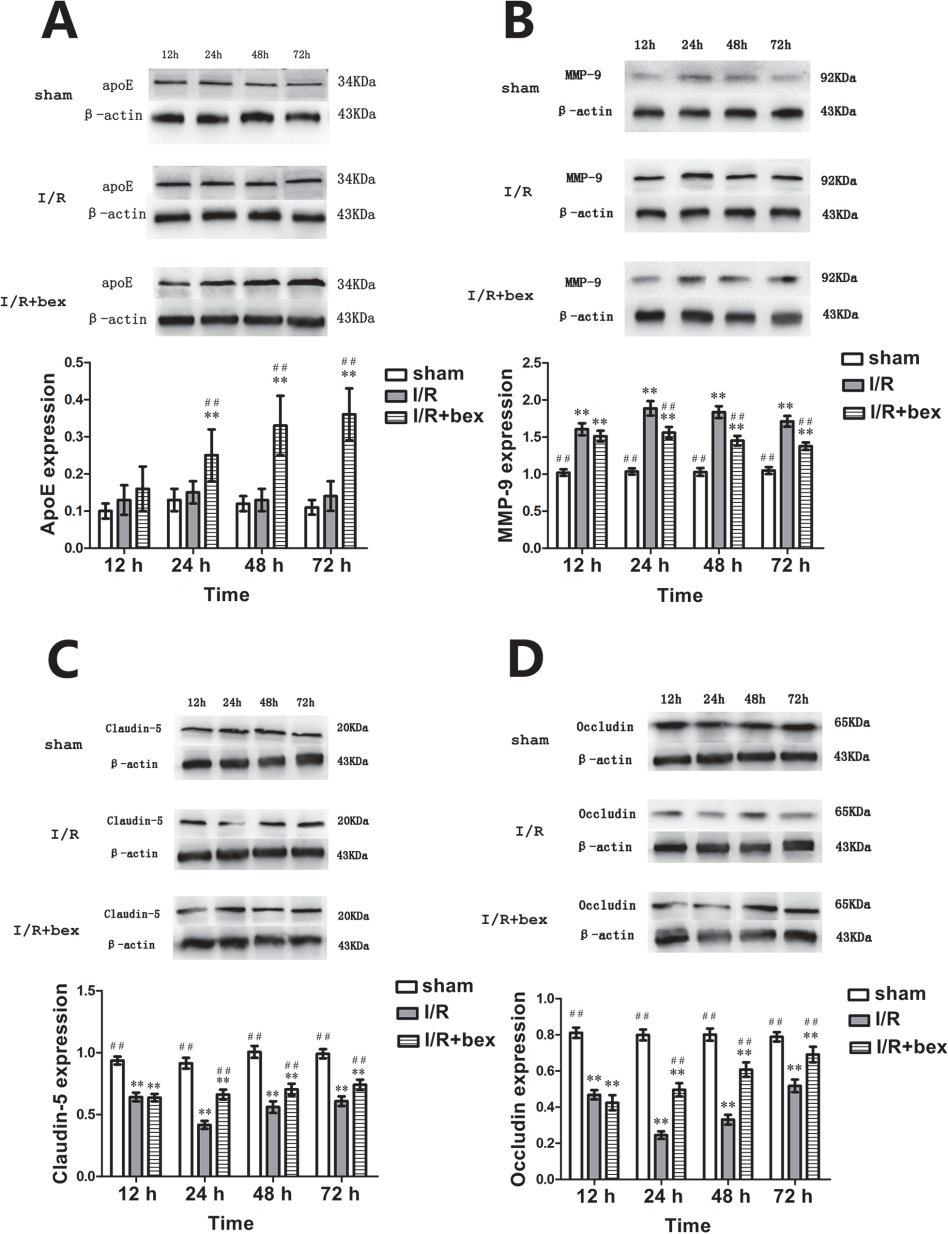
ApoE, MMP-9, Claudin-5, and Occludin Protein Expression. (A) ApoE (B) MMP-9, (C) claudin-5, and (D) occludin protein expression levels in the ischemic zone of the rat brain were determined by Western blotting and densitometric analysis with β-actin used as an internal control. Values are expressed as means±SDs (n = 5 per group; *P<0.05 versus the sham group, **P<0.01 versus the sham group; #*P*<0.05 versus the I/R group, and ##*P*<0.01 versus the I/R group).

At 24 h, 48 h, and 72 h post-I/R, apoE expression was significantly increased in the I/R+bexarotene group as compared with the sham-operation and I/R groups (*P*<0.01; Fig 7A). At 12 h, 24 h, 48 h, and 72 h, MMP-9 protein expression was significantly increased in the I/R and I/R+bexarotene groups as compared with the sham-operation group (*P*<0.01) with the highest expression achieved at 24 h (Fig 7B). At 24 h, 48 h, and 72 h, MMP-9 protein expression in the I/R+bexarotene group was significantly lower than that in the I/R group (*P*<0.01), while there was no significant difference at 12 h (*P*>0.05; Fig 7B). Notably, these MMP-9 protein expression findings match the MMP-9 mRNA expression and activity findings stated above. At 12 h, 24 h, 48 h, and 72 h post-I/R, both claudin-5 and occludin protein expression were significantly decreased in the I/R and I/R+bexarotene groups as compared with the sham-operation group (*P*<0.01) with the lowest expression achieved at 24 h (Fig 7C and 7D). At 24 h, 48 h, and 72 h post-I/R, both claudin-5 and occludin protein expression in the I/R+bexarotene group were significantly higher than those in the I/R group (*P*<0.01), while there was no significant difference at 12 h (*P*>0.05; Fig 7C and 7D).

## DISCUSSION

In this present study, we demonstrated that bexarotene administration significantly reduces brain water content and BBB disruption as evidenced by EB staining and Omniscan extravasation after I/R, supporting the neuroprotective properties of this drug. We also found that bexarotene treatment reduced the expression and activity of MMP-9, increased claudin-5 and occludin expression, and promoted apoE over-expression after I/R. These results suggest that the therapeutic effects of bexarotene in modulating BBB integrity post-I/R injury may be associated with the drug’s effect on apoE expression, which has been shown to downregulate MMP-9 expression and upregulate claudin-5 and occludin expression[7,8].

The BBB—which is formed by specialized endothelial cells of cerebral blood vessels—selectively restricts diffusion of compounds into the brain and is an important structure for maintaining cerebral homeostasis and proper neuronal function[9]. Tight junctions between the specialized endothelial cells that maintain the BBB’s integrity consist of integral membrane proteins (e.g., occludin, claudins, and junctional adhesion molecules (JAMs)) as well as cytoplasmic scaffolding proteins (e.g., zonula occludens (ZO) proteins) and actin cytoskeleton-associated proteins[9]. Disruption of these tight junctions has been positively associated with MMP-9 activity, and the tight junction proteins claudin-5 and occludin have been shown to be degraded by MMP-9 through cleavage of portions of their extracellular domains [10,11,12].

With respect to MMP-9’s role after cerebral ischemia, considerable research has proven that MMP-9 is up-regulated and activated after ischemic stroke and in animal models of I/R [13,14,15,16,17,18]. Here, we demonstrated that MMP-9 is significantly up-regulated and activated around 12 h post-I/R and achieves its climax at 24 h post-I/R. These changes in MMP-9 expression and activity coincided with an increase in BBB permeability and degradation of claudin-5 and occludin, suggesting that increases in MMP-9 activity and expression play a key role in promoting BBB permeability post-I/R. Moreover, we revealed that bexarotene treatment reduced the disruption of the tight junction via increasing claudin-5 and occludin protein expression after 24 h post-I/R, reduced the expression and activity of MMP-9 after 24 h post-I/R, and attenuated the increase in BBB permeability after 24 h post-I/R. Notably, the timing of the increases in claudin-5 and occludin protein expression observed with bexarotene treatment (after 24 h post-I/R) coincided with the timing of the reductions in the expression and activity of MMP-9 (after 24 h post-I/R) and the improvement in BBB permeability (after 24 h post-I/R), suggesting that bexarotene’s positive effects on BBB permeability post-I/R injury works through MMP-9-mediated upregulation of claudin-5 and occludin expression.

ApoE is the major apolipoprotein that maintains lipid homeostasis in the human brain and is a known neuroprotective factor with anti-inflammatory, anti-oxidative, and anti-excitotoxity benefits [19,20,21]. Extensive research has revealed that apoE deficiency increases BBB permeability in vivo and in vitro [7,22,23]. For example, apoE-deficient mice display more severe brain edema, higher BBB permeability, and worse cognitive impairment after brain injury [23,24,25], while exogenous intraventricular infusion of apoE displays significant therapeutic effects after cerebral ischemia injury [26]. Mechanistically, astrocyte-secreted apoE2 and apoE3 have been shown to suppress the pro-inflammatory cyclophilin A (CypA)-nuclear factor-κB (NF-κB)-MMP-9 pathway, a pathway which produces MMP-9-mediated BBB degradation[7]. Consistent with this view, a recent study by Zheng et al. demonstrated that ApoE deficiency in mice (ApoE−/−) upregulates MMP-9 expression, downregulates claudin-5 and occludin expression, and increases BBB permeability[8].

On this basis, therapeutic delivery of apoE to the brain would show promise in treating post-ischemic brain injury. However, intact apoE holoprotein (34 kDa) is too large to cross the BBB; fortunately, bexarotene (348.48 Da) is small enough to cross the BBB, and as a RXR agonist, is able to upregulate apoE expression [5,27]. In this study, we demonstrated that bexarotene administration started to non-significantly upregulate apoE expression after 12 h post-I/R and reached significant upregulation after 24 h. Notably, the timing of the significant apoE upregulation (after 24 h post-I/R) coincided with the improvement in BBB permeability (after 24 h post-I/R), reductions in the expression and activity of MMP-9 (after 24 h post-I/R), and claudin-5 and occludin upregulation (after 24 h post-I/R). Based on Bell et al.’s and Zheng et al.’s work[7,8], these combined findings suggest that bexarotene’s positive effects on BBB permeability post-I/R injury works through upregulating apoE’s suppression of the CypA-NF-κB-MMP-9 pathway. Further research on the mechanism(s) underlying bexarotene’s action on MMP-9, claudin-5, and occludin in the BBB post-I/R injury is required to validate this hypothesis.

In conclusion, although bexarotene is an established treatment for cutaneous T-cell lymphoma, its neuroprotective effects in a wide variety of brain diseases (i.e., Alzheimer's disease [5], Parkinson's disease [28], schizophrenia [29], and epilepsy [30]) have gathered increasing attention. Here, we showed that bexarotene treatment decreases BBB permeability in rats with cerebral I/R injury. This effect may be due in part to bexarotene’s upregulation of apoE expression, which has been previously shown to reduce BBB permeability through suppressing MMP-9-mediated degradation of the tight junction proteins claudin-5 and occludin. These results present a novel use for this old drug and may reveal a promising new treatment for cerebral ischemia patients.
